# Influence of Bisphosphonate Treatment on Medullary Macrophages and Osteoclasts: An Experimental Study

**DOI:** 10.1155/2012/526236

**Published:** 2012-09-13

**Authors:** Natalia Daniela Escudero, Patricia Mónica Mandalunis

**Affiliations:** Histology and Embryology Department, School of Dentistry, University of Buenos Aires, Marcelo T de Alvear 2142 1**º** piso sector A, (C1122AAH) Ciudad Autónoma de Buenos Aires, C1122AAH Buenos Aires, Argentina

## Abstract

Nitrogen-containing bisphosphonates are widely used for treating diverse bone pathologies. They are anticatabolic drugs that act on osteoclasts inhibiting bone resorption. It remains unknown whether the mechanism of action is by decreasing osteoclast number, impairing osteoclast function, or whether they continue to effectively inhibit bone resorption despite the increase in osteoclast number. There is increasing evidence that bisphosphonates also act on bone marrow cells like macrophages and monocytes. The present work sought to evaluate the dynamics of preosteoclast fusion and possible changes in medullary macrophage number in bisphosphonate-treated animals. Healthy female Wistar rats received olpadronate, alendronate, or vehicle during 5 weeks, and 5-bromo-2-deoxyuridine (BrdU) on day 7, 28, or 34 of the experiment. Histomorphometric studies were performed to study femurs and evaluate: number of nuclei per osteoclast (N.Nu/Oc); number of BrdU-positive nuclei (N.Nu BrdU+/Oc); percentage of BrdU-positive nuclei per osteoclast (%Nu.BrdU+/Oc); medullary macrophage number (mac/mm^2^) and correlation between N.Nu/Oc and mac/mm^2^. Results showed bisphosphonate-treated animals exhibited increased N.Nu/Oc, caused by an increase in preosteoclast fusion rate and evidenced by higher N.Nu BrdU+/Oc, and significantly decreased mac/mm^2^. Considering the common origin of osteoclasts and macrophages, the increased demand for precursors of the osteoclast lineage may occur at the expense of macrophage lineage precursors.

## 1. Introduction

Bisphosphonates, especially nitrogen-containing bisphosphonates, are the first-choice drugs in the pharmacological treatment of osteoporosis and other less prevalent bone pathologies. It is well documented that these anti-catabolic drugs exert their action by partly inhibiting bone resorption caused by osteoclasts, either by decreasing the number of osteoclasts, altering recruitment, and/or stimulating apoptosis, [1–7], after which the apoptotic remains are phagocytosed by neighboring macrophages in bone marrow microenvironment. Nevertheless, there are reports indicating that the number of osteoclasts remains unchanged in spite of the significant increase in bone volume [8, 9]. Moreover, a number of studies including our research group have observed a significant increase in the number of osteoclasts [10–18]. More recently, patients treated with alendronate were found to exhibit large osteoclasts, with peculiar morphological features, termed “giant osteoclasts”, whose formation, lifespan, and potential risk to patients remain unknown [19]. Similar findings from experimental studies in animals are scarce [16, 17, 20]. In addition, it has been reported that macrophages and monocytes are affected by bisphosphonate administration and the acute phase of the adverse reaction as well as the antitumor effects could be associated with the action of bisphosphonates on these cells [21]. 

Furthermore, it has been posited that the alteration of macrophages may play a role in the development of ONJ [22]. Preosteoclasts and macrophages have a common precursor: monocytes. The finding of hypernucleated osteoclasts in bisphosphonate-treated patients [19, 23] and in experimental animal models [16, 17, 20], has drawn attention to the dynamics of hypernucleated osteoclast formation and to the question whether macrophages are affected due to an increase in monocyte differentiation to the osteoclastic lineage. Based on the above, the aim of the present study was to assess the effect of bisphosphonates on macrophages and osteoclasts* in vivo*.

## 2. Materials and Methods

### 2.1. Experimental Design

**Figure 1:**
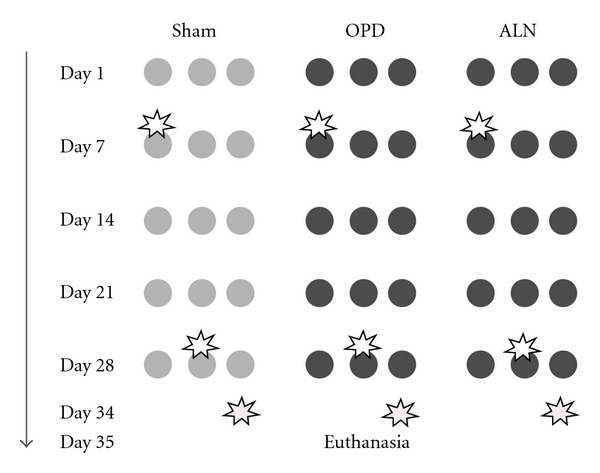
Experimental Design. Female Wistar rats were used, divided into three groups: Sham, OPD, and ALN. Each one of the groups was divided into three subgroups and the columns of circles indicate these subsets into which the animals in each group were divided. The rows show weekly administration of vehicle (light gray) or 0.3 mg/kg of the corresponding bisphosphonate olpadronate or alendronate (dark gray). The star indicates the time of administration of the single dose of BrdU, that is, days 7, 28, or 34 of the experiment. All animals were euthanized on day 35 of the experiment.

Twenty female Wistar rats, aged 2 months, 170 ± 10 gr body weight, were used throughout. Housing conditions included galvanized wire cages, five animals per cage, 21–24°C temperature, 52–56% humidity and 12-12 hrs light-dark cycles. The Guide for the Care and Use of Laboratory Animals (NRC 1996) was observed. The animals had free access to water and food (standard rat-mouse diet, Cooperación, Argentina) containing 23% protein, 1–1.4% calcium and 0.5–0.8% phosphorous. The animals were divided into three groups: two experimental and one sham. The experimental groups were i.p. injected with 0.3 mg/kg/week of monosodic olpadronate (OPD) and alendronate (ALN) (Gador SA), respectively, during 5 weeks; the dose was adjusted to body weight weekly. The sham group received an equal dose of saline solution during the same time interval. In addition, all animals received a single i.p. dose of 100 mg/kg body weight of 5-Bromo 2-deoxyuridine (BrdU). The animals in each of the groups were assigned to one of three subsets and injected with BrdU on day 7, 28, or 34 (i.e., one month, one week, or one day prior to euthanasia, resp.) (Figure 1). Euthanasia was performed on day 35 by administering acepromazine in a dose of 0.5 mg/kg bw (Acepromacina, Holliday Laboratories), ketamine 40 mg/kg bw (Ketamina 50, Holliday Laboratories), sodium pentobarbital 50 mg/kg bw and diphenylhydantoin sodium 6 mg/kg bw (Euthanyle, Brouwer Laboratories), all administered via i.p. After euthanasia left, femurs were resected.

### 2.2. Immunohistochemistry

The femurs were fixed in PBS-formalin at pH 7.2 and 4°C for 48 hrs and then decalcified in EDTA at the same temperature and pH. They were then dehydrated in ethyl alcohol (Biopack Argentina), clarified in xylol (Biopack Argentina) and embedded in paraffin to obtain longitudinal sections of the distal epiphysis. Two histologic sections were obtained from each femur; one was processed for immunohistochemical (IHC) detection of ED1 to detect cells of the phagocytic mononuclear system (macrophages, preosteoclasts, and osteoclasts belong to the phagocytic mononuclear system, so they express ED1 in the surface of their lysosomes), and the other was processed for immunohistochemical detection of BrdU to determine the number of stained nuclei in the osteoclasts.

For IHC detection of ED1, the sections were rehydrated in ethyl alcohol and washed with PBS 0.02 M (DakoCytomaton S3024). Antigen retrieval was performed with 0.1% trypsin in tris-maleate (Sigma-Aldrich, T0303-1G) at pH 7 and 37°C for 10 minutes. Following antigen retrieval, the sections were washed with PBS and endogenous peroxidase was blocked with 3% hydrogen peroxide (Biopack Argentina) in PBS for 10 minutes. The sections were incubated with mouse anti-rat ED1 monoclonal antibody (MAB 1435, Chemicon International Inc.) diluted 1 : 450 in PBS in a humidity chamber at 4°C overnight, following the manufacturer's instructions. 

For IHC detection of BrdU, the sections were dehydrated in ethanol and methanol (both Biopack Argentina). Endogenous peroxidase was then blocked with 1% hydrogen peroxide in methanol for 30 minutes. After washing with distilled water, antigen retrieval was performedbytwo, 3-minute cycles in 0.01 M citrate buffer in amicrowaveoven. Following antigen retrieval, the sections were allowed to cool to room temperature and unspecific reactions were blocked by immersion in 0.1% BSA (Sigma Aldrich) in PBS for 1 hour. The sections were incubated with the primary antibody reagent (1 : 100 in PBS with 1% BSA and 0.09% sodium azide at pH 7.6) (AM 247-5 M Biogenex) at room temperature in a humidity chamber for 30 minutes. 

After incubation with the primary antibody, anti-ED1 or anti-BrdU, the sections were washed with PBS and incubated with biotinylated antibody (BioGenex) for 1 hr, washed with PBS, and incubated with streptavidin-peroxidasecomplex. The sections were revealed with Diaminobenzidine(DAB) for 7 to 10 minutes (SK 4100, Vector Laboratories Inc), contrasted with hematoxylin, dehydrated, and mounted with Canada balsam. All sections for ED1 IHC detection were processed simultaneously, as BrdU sections.

### 2.3. Histomorphometry

From each histological section stained for detection of ED1, 35 digital microphotographs at 1000X magnification were obtained using a photomicroscope (Axioskop 2, Carl Zeiss Jena, Germany), and the number of macrophages in the dyaphiseal bone marrow was determined using Image Pro Plus 6.1 computer software (Media Cybernetics). Mononuclear cells showing cytoplasmic expression of ED1 were diagnosed as macrophages. Diaphyseal bone marrow was used, distant from any bone surface, because mononuclear cells neighboring bone trabeculae and cortical bone could be either macropages or preosteoclasts. In addition, the distribution of ED1 in the cytoplasm of osteoclasts located near the surface of subchondral trabeculae was observed and evaluated using 1000X magnification. Cells with 2 or more nuclei, in close relation with bone surface were diagnosed as osteoclasts (preosteoclasts are mononucleated, so 2 nuclei already represents multinucleation, and only osteoclasts are multinucleated). 

Histologic sections stained for BrdU detection were examined by direct observation under a light-field microscope using 1000X magnification to evaluate osteoclasts in primary and secondary spongiosa. The following parameters were studied: number of nuclei (N.Nu/Oc), mean number of BrdU-positive nuclei per osteoclast (N.Nu BrdU+/Oc), and percentage of BrdU-positive nuclei per osteoclast (%Nu. BrdU+/Oc).

The results were analyzed using one-way ANOVA or the Kruskal Wallis test accordingly, and the Bonferroni post hoc test; in order to study dependence between the number of nuclei per Oc and number of macrophages, Spearman's rank correlation was used. *p* values below 0.05 were considered significant.

## 3. Results

**Figure 2:**
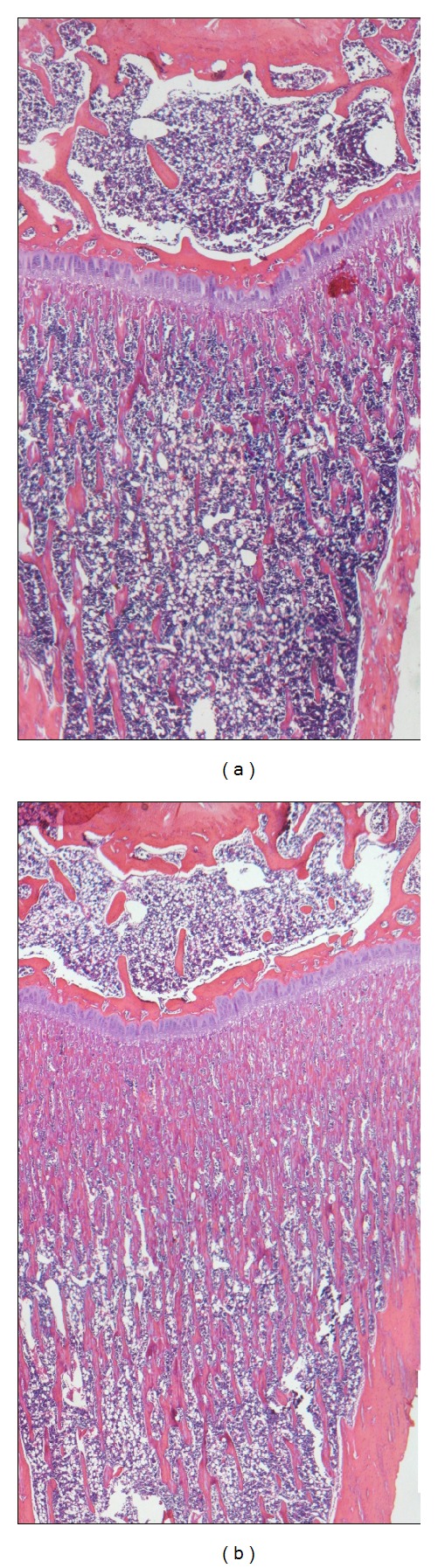
Microphotographs of hematoxylin-eosin-stained histological sections of distal tibia. Animals treated with BPs showed a larger number of subchondral trabecualae (b) compared to sham (a).

The effect of BPs on bone volume in tibia are illustrated in Figure 2.

**Figure 3:**
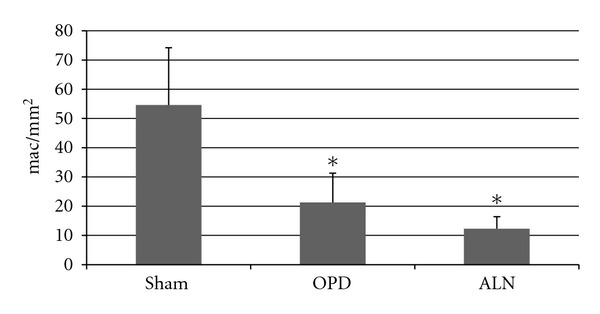
Number of macrophages in diaphyseal bone marrow. The number of macrophages, measured using IHC ED1 detection, was significantly lower in bisphosphonate-treated animals compared to sham (*: Anova *p* < 0.01 compared to sham).

**Figure 4:**
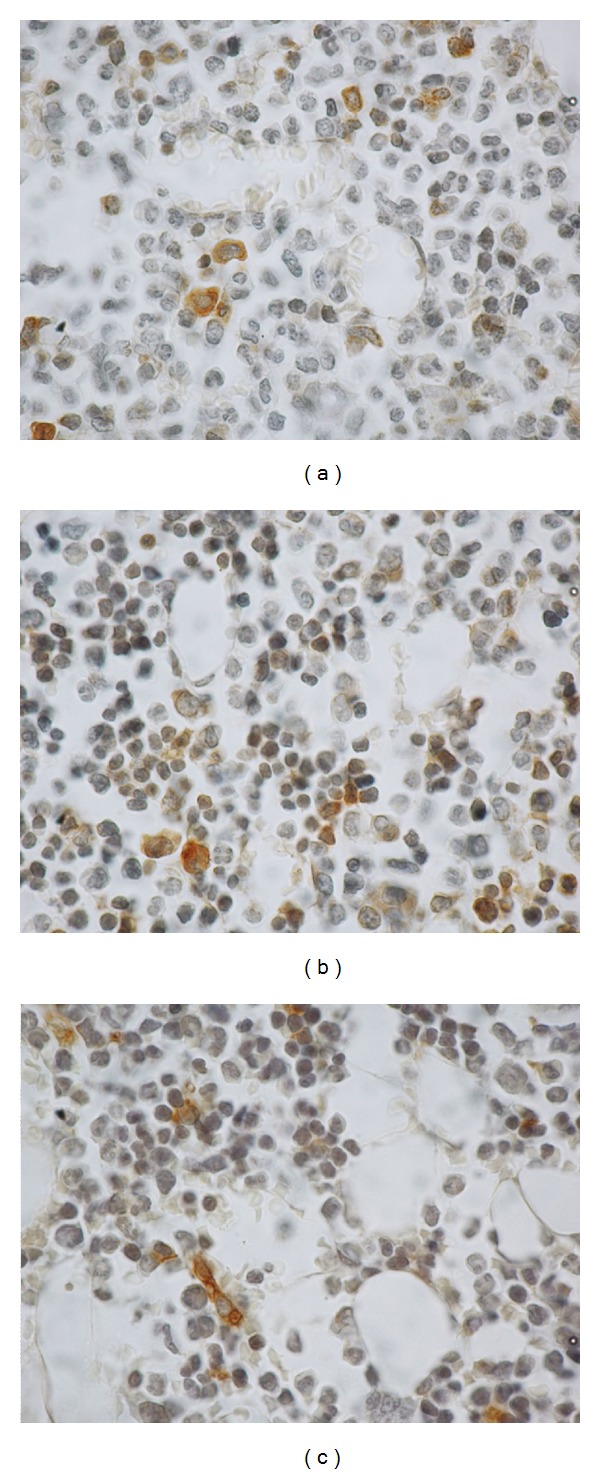
Macrophages in diaphyseal bone marrow. Microphotographs of histologic sections with ED1 immunohistochemical detection and hematoxylin-counterstain. Brown cells correspond to positive cells (macrophages). (a) shows a microphotograph of a sham animal, (b) an olpadronate-treated animal and (c) and alendronate-treated one. (b) and (c) show a lower number of positive cells.

 Sections stained immunohistochemically for detection of ED1 corresponding to bisphosphonate-treated animals showed a decrease in the number of macrophages per mm^2^: sham 54.6 ± 19.6 mac/mm^2^, OPD 21.3 ± 10 mac/mm^2^, ALN 12.3 ± 4.1 mac/mm^2^; Anova *p* < 0.01 (Bonferroni test: sham versus OPD and sham versus ALN) (Figures 3 and 4).

**Figure 5:**
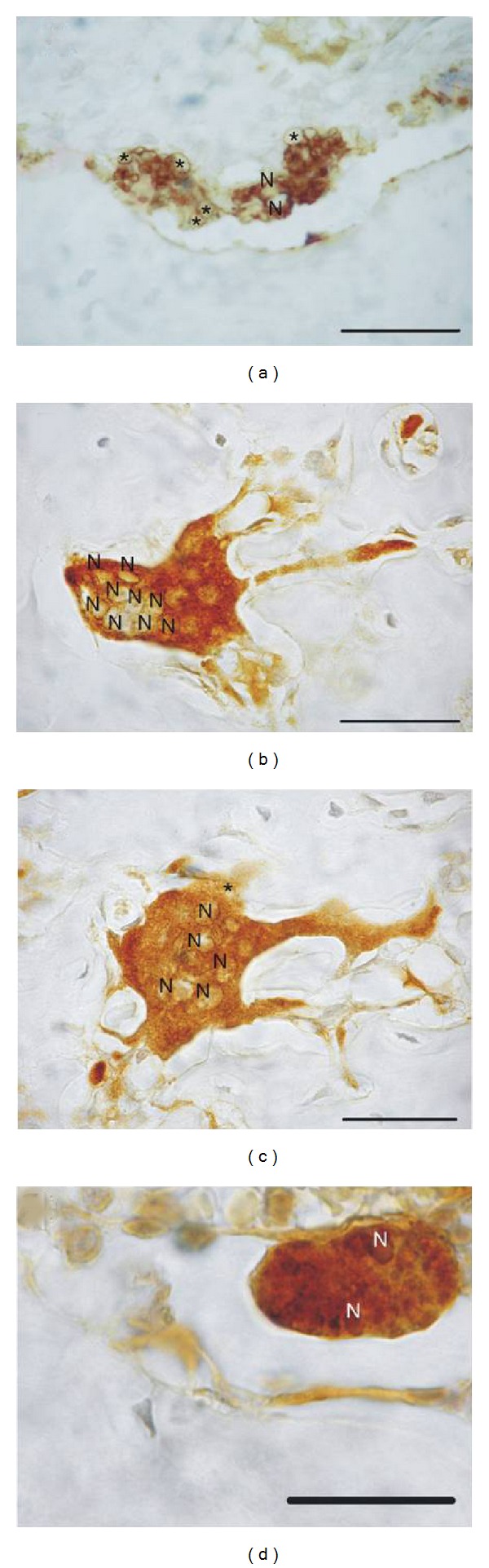
Microphotographs of osteoclasts stained for IHC detection of ED1. Microphotograph (a) shows the distribution pattern of the marker in the cytoplasm of osteoclasts of sham group sections; the light areas indicate abundant vesicles (*). (b) and (c) show osteoclasts corresponding to olpadronate and alendronate-treated animals, respectively. The cytoplasm exhibits a homogenous distribution pattern of ED1. (d) shows an apoptotic osteoclast staining intensely positive for ED1. N: nucleus. The bar represents 50 microns.

Evaluation of IHC expression of ED1 by osteoclasts showed all osteoclasts were ED1-positive, regardless of the treatment and osteoclast features. Even those exhibiting morphological features compatible with apoptosis were found to be ED1-positive (Figure 5(d)). Cytoplasmic expression varied in intensity and distribution pattern in the cytoplasm, and in some cases was found in some sectors only. Sham osteoclasts showed marked vacuolization whereas osteoclasts corresponding to bisphosphonate-treated animals exhibited a more homogenous distribution pattern (Figures 5(a), 5(b) and 5(c)). Large hypernucleated osteoclasts were found in the latter groups, and though most were detached from bone trabeculae they presented some active sectors superficially associated with an erosive bone surface. The presence of osteoclasts with long cytoplasmic projections extending through tubular resorption cavities in the bone matrix was a characteristic finding (Figures 5(b) and 5(c)).

 As regards BrdU, because this marker is incorporated into the cell nucleus during the S period of the cell cycle (prior to mitosis), osteoclasts of animals receiving BrdU on day 34 (24 hr before euthanasia) exhibited intense nuclear staining (when positive). The intensity of IHC staining for BrdU in histologic sections corresponding to animals receiving BrdU on day 28 of the experiment (one week prior to euthanasia) was similar to that observed on day 34. Both administration times were useful to assess recruitment of osteoclast precursors and their differentiation into multinucleated osteoclasts (taking into account that osteoclasts originate from fusion of posmitotic precursors). Nuclear staining in histologic sections of animals receiving BrdU on day 7 of the experiment, which was performed mainly to observe changes in osteoclast lifespan, was not as intense as that observed in sections corresponding to animals receiving BrdU on day 28 or 34 and it is in agreement with the fact that the intensity of BrdU staining is inversely proportional to the number of mitosis of the monocytic precursor in bone marrow.

**Figure 6:**
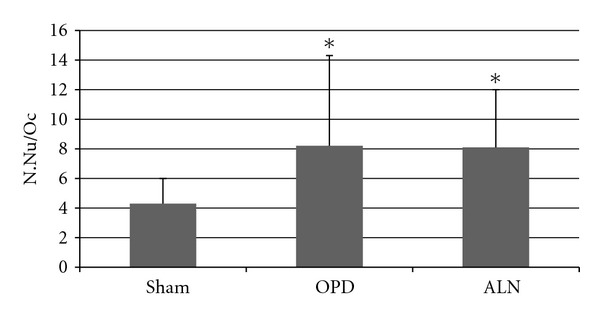
Mean number of nuclei in osteoclasts. The mean number of nuclei per osteoclast was evaluated in BrdU-detection sections, was significantly higher in bisphosphonate-treated animals compared to sham (*: Kruskal Wallis *p* < 0.01). Both positive and negative nuclei were counted to assess this parameter.

**N.Nu/Oc**

sham 4.3 ± 1.7 Nu/Oc, *n* = 7 animals; OPD 8.2 ± 6.1 Nu/Oc, *n* = 7 animals; ALN 8.1 ± 3.9 Nu/Oc, *n* = 7 animals, Kruskal Wallis *p* < 0.01, Bonferroni test: sham versus OPD and ALN, 825 Ocs were assessed in the three groups (Figure 6). Taking into account that number of nuclei is directly associated with osteoclast size, it follows that osteoclasts of bisphosphonate-treated animals were larger than those of sham animals.

**N.Nu BrdU+/Oc**

**Figure 7:**
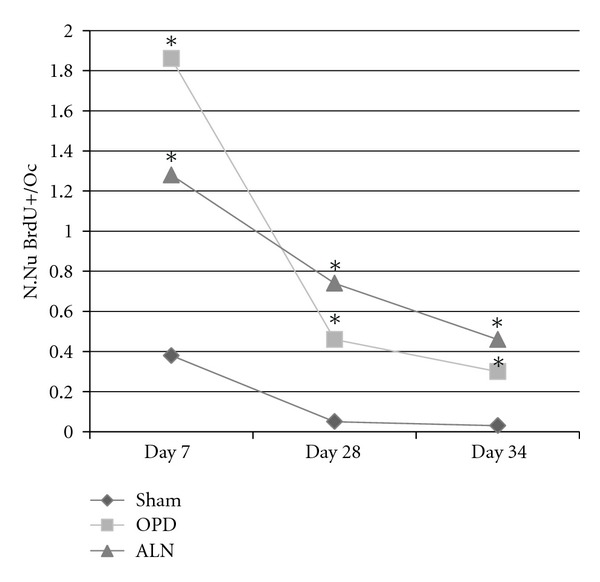
The number in BrdU-positive nuclei per osteoclast was greater in bisphosphonate-treated animals at all three BrdU administration times (*: Kruskal Wallis *p* < 0.001 for days 7 and 34, *p* < 0.05 for day 28). Standard deviations are not represented because of high dispersion, which produces lines superposition (see data on text).

**Figure 8:**
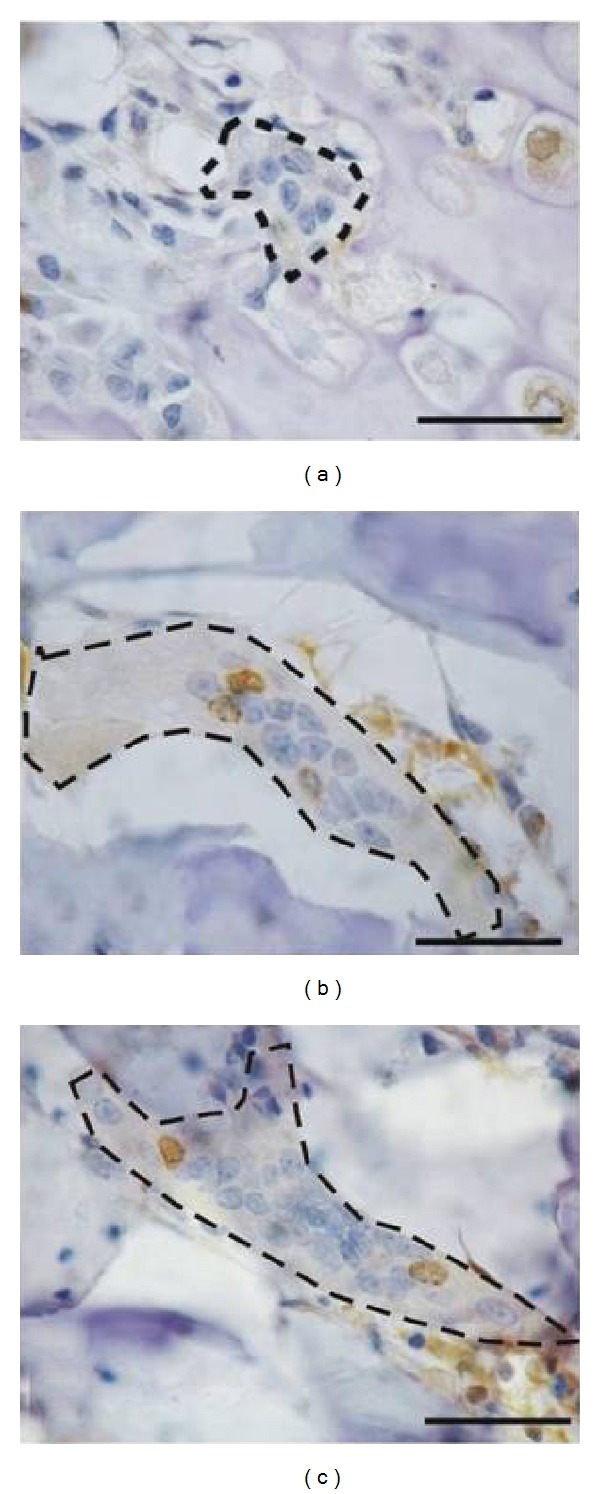
Microphotographs of osteoclasts stained immunohistochemically for BrdU. (a): dotted line: osteoclasts corresponding to the sham group showing 8 nuclei, none of which were BrdU-positive. (b): osteoclast corresponding to an olpadronate-treated animal showing at least 15 nuclear profiles, 3 of which were BrdU-positive. (c): osteoclast corresponding to an ALN-treated animal showing 2 BrdU-positive nuclei out of more than 25. The bar represents 50 microns.

Both groups of bisphosphonate-treated animals receiving BrdU on day 7 of the experiment showed a greater number of stained nuclei as compared to shams (sham 0.38 ± 0.68 Nu+/Oc *n* = 70 Ocs, OPD 1.86 ± 2.32 Nu+/Oc *n* = 75 Ocs, ALN 1.28 ± 2.18 Nu+/Oc *n* = 80 Ocs, Kruskal Wallis *p* < 0.001, Bonferroni sham versus OPD and sham versus ALN). The same trend was observed in animals receiving BrdU on day 28 (sham 0.05 ± 0.22 Nu+/Oc *n* = 40 Ocs, OPD 0.46 ± 0.95 Nu+/Oc *n* = 80 Ocs, ALN 0.74 ± 1.42 Nu+/Oc *n* = 120 Ocs, Kruskal Wallis *p* < 0.05, Bonferroni sham versus OPD and sham versus ALN) and in the subsets receiving BrdU on day 34 (sham 0.03 ± 0.18 Nu+/Oc *n* = 120 Ocs, OPD 0.3 ± 0.82 Nu+/Oc *n* = 120 Ocs, ALN 0.46 ± 0.86 Nu+/Oc *n* = 120 Ocs, Kruskal Wallis *p* < 0.001, Bonferroni sham versus OPD and sham versus ALN) (Figures 7 and 8).

**%Nu.BrdU+/Oc**

**Figure 9:**
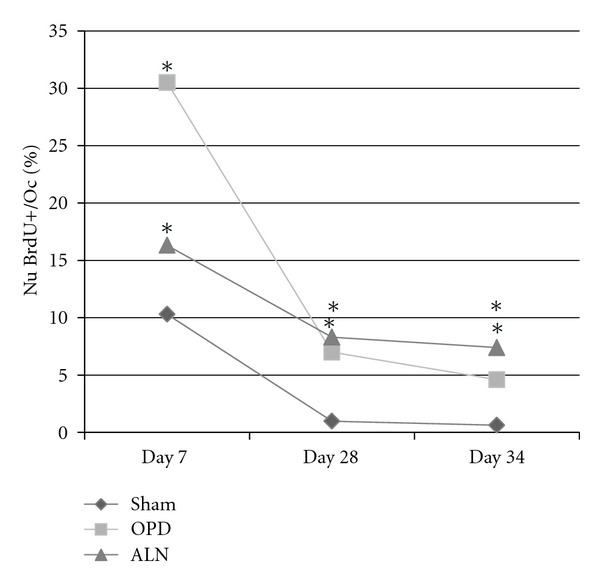
Percentage of BrdU positive-nuclei per osteoclast. The percentage of BrdU-positive nuclei per osteoclast was higher in the bisphosphonate-treated groups at all three BrdU administration times (day 7, 28, or 35 of the experiment corresponding to one month, one week, and one day before euthanasia resp.) (*Kruskal Wallis *p* < 0.05 compared to sham).

A significant increase in the percentage of positive nuclei per osteoclast (Oc) was observed in all BrdU subsets of bisphosphonate-treated animals: day 7: sham 10.3 ± 19.6%  *n* = 70 Ocs, OPD 30.5 ± 31.6%  *n* = 75 Ocs, ALN 16.3 ± 25.6  *n* = 105 Ocs; day 28: sham 0.98 ± 4.5%  *n* = 40 Ocs, OPD 7 ± 14.2%  *n* = 80 Ocs, ALN: 8.3 ± 15.3%  *n* = 120 Ocs; day 34: sham 0.63 ± 3.5%  *n* = 120 Ocs, OPD 4.6 ± 11.2%  *n* = 120 Ocs, ALN 7.4 ± 13.5%  *n* = 120 Ocs, Kruskal Wallis *p* < 0.05 in all cases, Bonferroni sham versus OPD and sham versus ALN at all three BrdU administration times (Figure 9).

**Correlation between the Number of Nuclei per Osteoclast and Number of Macrophages**

**Figure 10:**
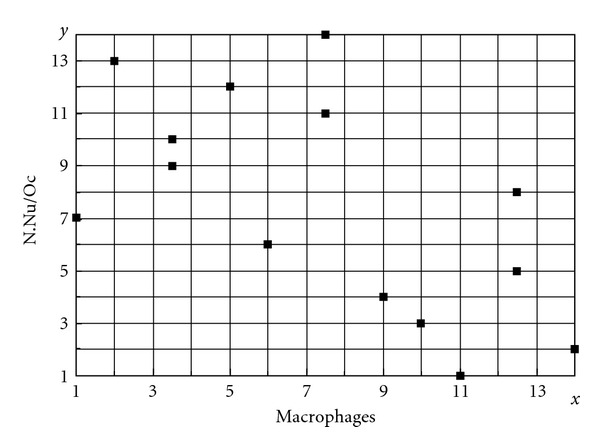
Number of nuclei per osteoclast and number of macrophages. These two variables showed a negative correlation (−0.6), as number of nuclei increased in BP-treated animals, the number of macrophages in bone marrow consequently decreased (*n* sham = 7, *n* BP-treated = 7). *p* < 0.05, Spearman's test.

An inverse correlation (−0.6) was found between N.Nu/Oc and mac/mm^2^ (Figure 10): as number of nuclei per osteoclasts increased in BP-treated groups, the number of macrophages decreased (Spearman's test *p* < 0.05).

## 4. Discussion

The results of the present work show that animals treated with both the bisphosphonates exhibited a significant decrease in the number of medullary macrophages as compared to shams. In their 1999 study on mice treated with the aminobisphosphonate AHBuBP, Nakamura et al. also observed a decrease in erythroblastic island macrophages [14]. Macrophages have multiple functions, and their decrease, therefore, can have a number of implications. One such function is the removal of apoptotic cells. In previous works in bisphosphonate-treated animals, we found an increase in the number of osteoclasts as well as an increase in the number of apoptotic osteoclasts [17]. The question thus arises whether the increase in the number of apoptotic osteoclasts observed in histologic sections is caused only by the effect of bisphosphonates on osteoclasts, or whether apoptotic debris remain in the bone microenvironment over a more prolonged period of time due to a deficiency in phagocytosis. As osteoclast size is directly associated with the number of precursors that fuse to form multinucleated osteoclasts, it can be posited that those presenting a large number of nuclei were, in turn, large osteoclasts; removal of such large osteoclasts may contribute to the inefficient clearance of debris.

Macrophages also have an immune function, and its impairment could thus be associated with the development of osteonecrosis of the jaw, as has been suggested previously [22, 24].

The finding of a large number of nuclei in BrdU-stained sections is in agreement with reports by other authors [19] who found “giant” osteoclasts containing a large number of nuclei and that were detached from the bone surface. Our observation of giant osteoclasts coexisting with normal-appearing osteoclasts in bisphosphonate-treated animals is also in keeping with the aforementioned report. The coexistence of osteoclasts similar in size to controls with other larger osteoclasts is shown by the greater dispersion of the data on the number of nuclei found in both bisphosphonate-treated groups. Based on the observation of stained nuclei in bisphosphonate-treated animals receiving BrdU 24 hrs before euthanasia it can be inferred that not all giant osteoclasts detached from the bone surface are inactive forms that will remain inactive from a recruitment viewpoint, but rather may be found to be actively incorporating precursors. It cannot be concluded that these osteoclasts were significantly smaller and functionally normal at some previous stage. The finding of a greater number of nuclei in osteoclasts from bisphosphonate-treated animals is in agreement with previous *in vivo* studies in children with osteogenesis imperfecta treated with risedronate [23] and patients with osteoporosis treated with alendronate [19]. BrdU staining resulting from BrdU administration 24 hrs and 1 week before euthanasia showed that the number and percentage of stained nuclei were higher in both bisphosphonate-treated groups. Our results are in keeping with those of other authors who used BrdU [11] and tritiated thymidine [25]; the latter are the only studies reported in the literature using nuclei staining to evaluate osteoclast recruitment. Staining with BrdU administered 1 month before euthanasia was almost undetectable; the number and percentage of stained nuclei were higher in bisphosphonate-treated than sham animals and in the subset receiving BrdU on day 7 compared to the remaining subsets of the same group. The difference between bisphosphonate-treated and sham animals may be due to an increased fusion of osteoclast precursors caused by bisphosphonates, as found in groups receiving BrdU 24 hrs and 1 week prior to euthanasia. The difference among subsets of the same group may be because renewal of hematopoietic stem cells results in a greater number of stained precursors, though intensity of staining is proportionally lower to the number of mitosis of the hematopoietic stem cell. The main aim of administering BrdU at this experimental time (1 month before euthanasia) was to observe changes in osteoclast lifespan. Such changes could be a shortening of osteoclast longevity manifesting as fewer or lack of BrdU-positive nuclei compared to shams, or a lengthening of osteoclast lifespan made apparent by more intensely stained nuclei in bisphosphonate treated animals as compared to controls. Our study could not detect changes in osteoclast lifespan. The lack of differences indicates that the lifespan of osteoclasts was less than one month, that is, the administration time used to evaluate osteoclast lifespan, and would explain why less-marked changes might not have been detected in our experimental design. The use of shorter administration times of BrdU that allow assessing less extreme changes in osteoclast lifespan may prove useful for designing future studies. 

Considering that osteoclasts with 30 or more nuclei had 1 or 2 positive nuclei when BrdU was administered 24 hrs prior to euthanasia, it follows that the time course of osteoclast formation in this case could not have been less than 15 days, provided that the rate of precursor fusion remained constant. The time course of normal osteoclast formation, however, is no longer than 4 to 6 days [26]. Hence, although no changes in osteoclast lifespan were detected, it can be concluded that the time for fusion of osteoclasts with additional mononuclear progenitors is longer in bisphosphonate-treated animals. In addition, as mentioned previously, some giant osteoclasts seemed to be partially active. It remains to be clarified whether in spite of having initiated resorption, fusion of these osteoclasts with precursors continues, thus increasing in size as they continue to attempt but fail to resorb bone matrix.

As to the rate of precursor recruitment, the sharper slope observed in bisphosphonate-treated animals from day 28 to day 34 of BrdU administration depicted in Figure 5 may be associated with a higher rate of precursor fusion. The greater number of stained nuclei observed in animals receiving BrdU on day 7 as compared with the subsets of the same group (i.e., the remaining BrdU administration times) is due to mitosis of the hematopoietic stem cell. 

It remains to be elucidated whether the decrease in the number of macrophages observed in the present study is due to a direct effect of bisphosphonates on these cells [21, 27] or whether monocytes, the precursors of macrophages and preosteoclasts, become committed to the osteoclast lineage at the expense of the macrophagic lineage [14] supporting our finding of negative correlation between number of nuclei and number of macrophages, also explaining the presence of hypernucleated, large osteoclasts. Further studies are necessary to determine if macrophage depletion is directly related to the increase in osteoclast number or if they are effects of bisphosphonates, independent one to another.

It is well documented that increased RANKL expression in the environment promotes differentiation of preosteoclasts to osteoclasts [28, 29]. In addition, previous findings reported by our group showed that nitrogen-containing bisphosphonates increase the number of megakaryocytes, which express RANKL, and thus might act as an additional source of RANKL in the bone microenvironment and contribute to precursor cells committing to the osteoclast lineage at the expense of the macrophagic lineage [17].
